# Effect of *Codonopsis pilosula* polysaccharide on the quality of sheep semen preservation at 4°C

**DOI:** 10.5713/ab.23.0258

**Published:** 2024-01-20

**Authors:** Yuqin Wang, Yanhong Zhao, Hua Chen, Tingting Lu, Rujie Yang, Xiuxiu Weng, Wanhong Li

**Affiliations:** 1 National Demonstration Center for Experimental Grassland Science Education, Key Laboratory of Grassland Livestock Industry Innovation, Ministry of Agriculture and Rural Affairs, College of Pastoral Agriculture Science and Technology, Lanzhou University, Gansu Lanzhou 730020, China; 2 Tianzhu Animal Breeding Research Institute, Gansu Wuwei 733000, China; 3 Wuwei Animal Husbandry and Veterinary Science Research Institute, Gansu Wuwei 733000, China; 4 Gansu Lantian Tonghe Co. Ltd., Gansu Wuwei 733000, China

**Keywords:** *Codonopsis pilosula* Polysaccharide, Motility, Sheep, Sperm

## Abstract

**Objective:**

This study aimed to investigate the effect of *Codonopsis pilosula* polysaccharide (CPP) on the motility, mitochondrial integrity, acrosome integrity rate, and antioxidant ability of sheep sperm after preservation at 4°C.

**Methods:**

Semen from healthy adult rams were collected and divided into four groups with separate addition of 0, 200, 400, and 1,000 mg/L CPP. Sperm motility was analyzed using the Computer-Assisted Semen Analysis software after preservation at 4°C for 24, 72, 120, and 168 h. Sperm acrosome integrity rate was analyzed by Giemsa staining at 24, 72, and 120 h, and mitochondrial membrane integrity was analyzed by Mito-Tracker Red CMXRos. The total antioxidant capacity (T-AOC) and malondialdehyde (MDA) content of spermatozoa were measured after 120 h of preservation.

**Results:**

The sperm viability and forward-moving sperm under 200 mg/L CPP were significantly higher than that in the control group at 72 h (61.28%±3.89% vs 52.83%± 0.70%, 51.53%±4.06% vs 42.84%±1.14%), and 168 h (47.21%±0.85% vs 41.43%±0.37%, 38.68%±0.87% vs 31.68%±0.89%). The percentage of fast-moving sperm (15.03%±1.10% vs 11.39%±1.03%) and slow-moving sperm (23.63%±0.76% vs 20.29%±1.11%) in the 200 mg/L group was significantly higher than control group at 168 h. The mitochondrial membrane integrity of the sperm in the group with 200 mg/L CPP was significantly higher than those in the control group after storage at 4°C for 120 h (74.76%±2.54% vs 65.67% ±4.51%, p<0.05). The acrosome integrity rate in the group with 200 mg/L (87.66%±1.26%) and 400 mg/L (84.00%±2.95%) was significantly higher than those in the control group (80.65%±0.16%) after storage for 24 h (p<0.05). CPP also increased T-AOC and decreased the MDA concentration after preservation at 4°C (p<0.05).

**Conclusion:**

Adding CPP could improve the T-AOC of sperm, inhibit lipid peroxidation, and facilitate semen preservation.

## INTRODUCTION

With the development of the sheep industry and breeding, artificial insemination technology is taking an increasingly important role in animal husbandry. Among them, semen preservation is one of the most critical steps of artificial insemination technology. Due to the violent content of unsaturated fatty acids in the plasma membrane of sperm, it is susceptible to oxidative damage during preservation [1]. Superoxide anions, peroxides, and reactive oxygen species (ROS) produced by sperm could cause lipid peroxidation reactions that severely damage the integrity of the acrosome structure, plasma membrane, and sperm DNA. Therefore, the addition of antioxidants to sperm preservation diluents has become a vital means of reducing oxidative damage to sperm, improving its high preservation efficiency, and prolonging its preservation time.

Academic studies on the beneficial effects of adding antioxidants on sperm preservation have been dominated by the increase in the activity of antioxidant enzymes in sperm. Eslami et al [2] found that adding idebenone to dilutions reduced ROS levels by increasing catalase (CAT) and superoxide dismutase (SOD) activity, thereby inhibiting lipid peroxidation. Idebenone improved ram sperm motility by reducing lipid hydroperoxide, malondialdehyde (MDA), and nitric oxide concentrations and increasing the activity of SOD and the total antioxidant capacity (T-AOC). Meanwhile, non-enzymatic antioxidants were directly added to improve the preservation of sheep semen. The aqueous extract of *Moringa oleracea* leaves, proanthocyanidins, and vitamin C could counteract free radicals and provide protection against oxidative stress by adding them [3–5].

Plant polysaccharides are also receiving increasing attention as antioxidants. Studies have shown that *Codonopsis pilosula* polysaccharide (CPP) could improve the body’s antioxidant capacity by reducing free radical levels and increasing the activity of antioxidant enzymes while enhancing immunity, antitumor activity, and other biological functions of the body. The ability of CPP to scavenge superoxide anions and hydroxyl radicals was positively correlated with the concentration of CPP [6]. Furthermore, CPP could increase the SOD and glutathione peroxidase activities in the blood of mice and decrease the MAD concentration of lipid peroxidation products [7]. On the basis of the above antioxidant properties, the effects of CPP on the ram sperm motility, sperm acrosome integrity, and antioxidant ability after semen preservation at 4°C were investigated in the present study.

## MATERIALS AND METHODS

### Main reagents

Analytically pure CPP, skim milk powder, soybean ovalbumin, and penicillin mixture (for cell culture) were purchased from Beijing Solarbio Science (Beijing, China). Fructose and glycerol were purchased from Sigma–Aldrich (Shanghai, China). Giemsa staining and Mito-Tracker Red CMXRos were purchased from Shanghai Beyotime Biotechnology (Shanghai, China). The T-AOC assay kit, MDA assay kit, and BCA protein assay kit were purchased from Nanjing Jiancheng Bioengineering Institute (Nanjing, China).

### Configuration of the dilution solution

Skim milk powder (50 g), soy lecithin (1.875 g), and fructose (6.25 g) were weighed into a 200 mL beaker and dissolved with thorough stirring, filtered, autoclaved to cool, and then transferred to a sterile 250 mL-volumetric flask; 2.5 mL of 100×penicillin + streptomycin solution was subsequently added. Distilled water was added to bring the volume up to 250 mL, resulting in the preparation of dilution I [8]. Sterile glycerol was added at a ratio of 37:3 (diluent I: glycerol) and mixed well to obtain the low-temperature base diluent. Lastly, precise amounts of CPP were then dissolved in 20 mL of low-temperature basal dilutions, and dilutions of sheep semen containing 0, 200, 400, and 1,000 mg/L of CCP were prepared at a low temperature. The semen dilutions were placed in a 35°C water bath for future use.

### Semen collection and dilution

The sheep semen used for the experiment was obtained from three healthy 2 to 3-year-old rams without germline diseases from Gansu Lantian Tonghe Agricultural Co. Ltd. Fresh semen was collected pseudo-vaginally, mixed, placed in 10 mL centrifuge tubes, and diluted with at a 1:8–10 ratio to achieve a sperm density of 3 to 5×10^8^ sperm/mL. The sperm was kept in a bubble box with 200 mL of warm water at 35°C and then stored in the refrigerator at 4°C. The ejaculates collected from the three rams were pooled and processed to balance the sperm contribution of each male and eliminate variability. The centrifuge tubes were gently shaken every 12 h to prevent sperm sedimentation from affecting sperm preservation [9].

### Evaluation of sperm motility

After being preserved at 4°C for 24, 72, 120, and 168 h, the sperm motility was analyzed with Computer-Assisted Semen Analysis software (Minitube, Tiefenbach, Germany). For each sample, 10 μL of semen was placed on a slide and covered with a coverslip. The motility parameters were observed with a 200× microscopic objective to determine the sperm motility in five fields of each sample.

### Evaluation of acrosome integrity rate

After being preserved at 4°C for 24, 72, and 120 h, 10 μL of sperm was aspirated and swabbed. After natural air drying, the slides were macerated in 4% paraformaldehyde solution for about 2 h, rinsed with distilled water, air dried, and then placed in Giemsa solution. Next, they were stained for 1 h, rinsed with distilled water, and air-dried. The morphological structure of sperm acrosomes on the slides was observed under 1,000× magnification, and the acrosome integrity rates were calculated as the rate of intact sperm acrosomes = several sperm with intact acrosomes/a total number of sperm ×100% [9].

### Evaluation of mitochondrial integrity

After the sperm was preserved at 4°C for 24, 72, and 120 h, 10 μL of Mito-Tracker Red CMXRos working solution and 10 μL of the stained sperm were added in a tube and mixed well. Subsequently, 10 μL of the stained sperm was placed on a slide after incubation at 37°C for 15 min. The integrity of the mitochondrial membrane potential was determined on the basis of different fluorescence of the sperm under a fluorescence microscope as follows: number of spermatozoa with red fluorescence/total number of spermatozoa ×100%.

### Effect of CPP on sperm antioxidant ability

Sperm was collected by centrifugation after being stored at 4°C for 120 h. It was broken by the freeze-thaw method, and the T-AOC and MDA concentrations in the spermatozoa were determined using the T-AOC assay kit and the MDA assay kit in accordance with the manufacturer’s instructions, respectively. T-AOC and MDA were normalized with the protein concentration.

### Statistics and analysis of data

The experimental data were expressed as mean±standard error of the mean and analyzed using IBM SPSS 22.0 (SPSS, Chicago, IL, USA). Statistical analysis was also performed using IBM SPSS 22.0 (SPSS, USA) for one-way analysis of variance and least significant difference method for multiple comparisons. p<0.05 indicated a significant difference.

## RESULTS

### Effect of CPP on sheep sperm motility after storage at 4°C

CPP significantly affected sperm motility after preservation at 4°C at different times (Table 1). Among them, the total sperm motility and forward-moving sperm under 200 mg/L CPP was significantly higher than that in the control group at 72, and 168 h (p<0.05). The percentage of fast-moving sperm and slow-moving sperm in the 200 mg/L group was significantly higher than control group at 168 h. The percentage of total sperm motility, forward-moving sperm and fast-moving sperm in 1,000 mg/L was also significantly higher than those in control group at 120 hours (p<0.05). After 168 h preservation, the percentage of forward-moving sperm was decreased just nearly under 30%. So we did not analyze the other index of sperm after 168 h preservation.

### Effect of CPP on the mitochondrial integrity of sheep spermatozoa after storage at 4°C

CPP significantly affected the sperm mitochondrial integrity after preservation at 4°C at different times (Table 2). Compared with control, 200 mg/L CPP significantly increased the sperm mitochondrial integrity at 24, 72, and 120 h (p< 0.05). At 120 h, the 1,000 mg/L CPP also significantly increased the sperm mitochondrial integrity compared with control group without CPP (p<0.05).

### Effect of CPP on the acrosome integrity rate of sheep spermatozoa after 4°C preservation

Compared with the control, 200 and 400 mg/L CPP significantly increased the sperm acrosome integrity rate (p<0.05) after preservation at 4°C for 24 h. However, no significant differences were found among the groups at 72 and 120 h (Table 3).

### Effect of CPP on the T-AOC and MDA of sheep spermatozoa after 4°C preservation

**Figure 1:**
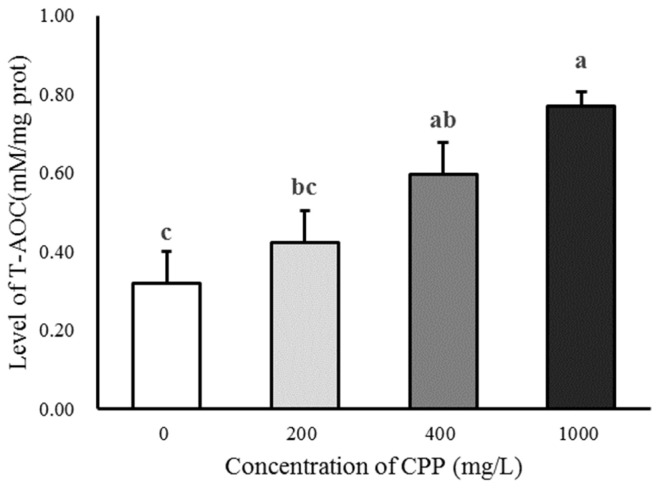
Effect of *Codonopsis pilosula* polysaccharide (CPP) on total antioxidant capacity (T-AOC). CPP could increase the content of T-AOC in sperm after 5 days of storage, and the T-AOC activity in 400 mg/L and 1,000 mg/L CPP groups was significantly higher than that in the control group (p<0.05). ^a–c^ Different letters indicate significant differences (p<0.05).

**Figure 2:**
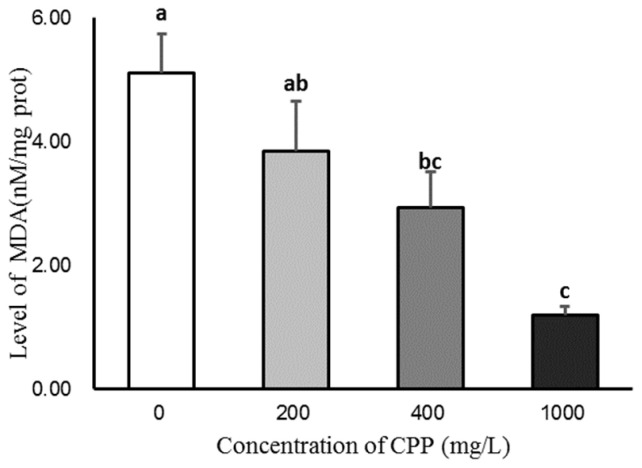
Effect of *Codonopsis pilosula* polysaccharide (CPP) on malondialdehyde (MDA). The content of MDA was decreased by the CPP in sperm after 5 days of storage, and the content of MDA in the 400 and 1,000 mg/L CPP groups was significantly lower than that in the control group (p<0.05). ^a–c^ Different letters indicate significant differences (p<0.05).

CPP could increase the content of T-AOC in sperm after 5 days of storage, and the T-AOC activity in 400 and 1,000 mg/L CPP groups was significantly higher than that in the control group (p<0.05; Figure 1). The content of MDA was decreased by the CPP in sperm after 5 days of storage, and the content of MDA in the 400 and 1,000 mg/L CPP groups was significantly lower than that in the control group (p<0.05; Figure 2).

## DISCUSSION

In semen preservation, the nutrients necessary for the metabolic process of sperm are provided exclusively by the diluent. Low temperatures slow down sperm motility and metabolism and prolong the time of sperm preservation *in vitro*. Although ROS can regulate sperm physiological functions and improve zona pellucida binding, their excessive production during sperm dilution, temperature reduction, and preservation at a low temperature of 4°C could cause severe damage to sperm DNA, plasma membrane, and mitochondrial structure and function, thereby shortening the duration of *in-vitro* preservation [10]. As the number of dead sperm increases during sheep sperm preservation, the activity of aromatic amino acid oxidase, one of the major enzymes responsible for the production of ROS in dead sperm, increases, leading to increased lipid oxidation of sperm and increased MDA concentration, which aggravates the damage to living sperm [11,12]. Previous studies also found that with increasing duration of sheep sperm preservation, sperm viability, and motility parameters decreased to varying degrees, and the T-AOC and SOD activity decreased significantly; the MDA concentration also increased significantly and was 2 to 5 times higher after 120 h of storage than after 24 h of storage, depending on dilution [9]. In the present work, sperm viability indicated a decreasing trend with increasing storage time, which is consistent with the above results. Sheep spermatozoa were stored at 4°C for 120 h and added with CPP to improve sperm viability. Many studies have shown that the fertility rate of artificial insemination of sheep sperm stored at low temperatures of 0.5°C could reach more than 60%, and the results are close to those of insemination with fresh sperm [13].

In this experiment, we added different concentration of CPP in the sperm dilution solution, to detect the values of T-AOC and MDA after 5 days, and found that their changes were consistent with the experimental conclusions of mice. In mice, CPP could significantly increase the activity of SOD and glutathione peroxidase, block the formation of free radicals and lipid peroxide, inhibit the production of lipid hydroperoxides, and thus decrease the MDA level to achieve antioxidant function [14]. *In vitro*, experiments also revealed that CPP could improve the antioxidant capacity of rat testicular mesenchymal cells by increasing the activity of glutathione peroxidase and decreasing the level of lipid peroxidation and MDA concentration in mesenchymal cells; the activity of CPP on SOD enzymes also increased with the extension of treatment time [15]. The intracellular superoxide anion is catalyzed by SOD and CAT to generate H_2_O and O_2_, which are then scavenged to maintain the balance of intracellular redox reactions and keep the reactive oxygen radicals at a level that is not harmful to the organism [16]. Jin et al [17] reported that CPP could achieve an antioxidant effect by increasing the activity of CAT [18,19]. Hydrogen peroxide has a great effect on tyrosine phosphorylation, but high concentrations of it could damage sperm DNA, inhibit the occurrence of sperm capacitation reactions, and affect the activity of acrosome enzymes [14]. In the present study, the addition of CPP preserved for 120 h significantly increased sperm T-AOC, inhibited the occurrence of lipid peroxidation and reduced the production of MDA.

The acrosomal enzymes in sperm acrosome play an important role in the lysis of the corona radiata and zona pellucida during fertilization, and premature release of acrosomal enzymes results in blocked sperm-ovum binding. In *in-vitro* sperm preservation, disruption of acrosomal integrity is one of the major factors leading to reduced sperm fertilization ability. However, the polyunsaturated fatty acids in the sperm plasma membrane play an important role in its integrity and sperm-oocyte fusion. When spermatozoa are attacked by ROS, these polyunsaturated fatty acids are easily lost by oxidation, consequently affecting plasma membrane fluidity, ion exchange processes, mass transfer reactions, and protease activity [15]. In this study, the addition of 200 mg/L CPP was found to improve sperm quality, likely due to its ability to bind to ROS and exert antioxidant effects. CPP, a key component of codonopsis polysaccharides, is composed of a galactolic acid backbone region and a rhamnose galactonic acid I side chain region, which contributes to its bioactivity [20]. The study also revealed a positive correlation between CPP concentration and the generation of ROS and hydrogen peroxide in the free radical production system, suggesting that certain components of CPP, such as glycopeptides and binding proteins, possess strong antioxidant properties [21]. By incorporating CPP into the dilution solution, the study demonstrated its effectiveness in protecting sperm from early acrosomal reaction and enhancing acrosomal membrane integrity, leading to improved sperm preservation quality. This antioxidant function may be attributed to the large number of hydroxyl groups present in the structure of CPP, which can bind to ROS due to its high content of sugar molecules like galactose, rhamnose, and galacturonic acid. Therefore, in the present study, adding CPP to the dilution solution effectively protected sperm from early acrosomal reaction and improved acrosomal membrane integrity, which contributed to the improvement of sperm preservation quality. The mitochondria are the energy center of sperm, providing sufficient energy for its metabolism and motility through oxidative phosphorylation to produce adenosine triphosphate and maintain its motility [22]. In the present study, the integrity of the mitochondria and acrosomes in the 200 mg/L CPP group was significantly higher than that in the control group when sperm was preserved for 24 to 120 h, consistent with the study that revealed CPP could improve sperm motility. Similar conclusions were drawn in the study on the effect of lipopolysaccharide-binding protein and Astragalus polysaccharide on sheep sperm preservation [9].

## CONCLUSION

CPP could promote the preservation of ram semen by improving the antioxidant capacity of the spermatozoa and inhibiting lipid peroxidation. Thus, adding 200 mg/L CPP to 4°C preservation solution of sheep spermatozoa could increase the protective efficacy and significantly improve sperm viability and mitochondrial membrane integrity after 5 days of preservation.

## Figures and Tables

**Table 1:** Effect of different concentrations of *Codonopsis pilosula* polysaccharide (CPP) on the motility of spermatozoa after preservation

Time	CCP conc. (mg/L)	Percentage of total motile sperm (%)	Percentage of forward-moving sperm (%)	Percentage of fast-moving sperm (%)	Percentage of slow-moving sperm (%)	Percentage of partially motile sperm (%)
0 h		82.05±1.22	68.42±1.67	41.73±2.89	26.58±4.31	13.63±0.63
24 h	0	65.18±2.01	59.19±1.97	17.61±4.30^b^	41.35±3.84^a^	6.00±0.34^c^
24 h	200	65.15±1.51	58.39±0.30	22.22±7.47^ab^	36.17±7.22^ab^	6.77±1.32^bc^
24 h	400	64.55±1.2	54.3±1.47	35.05±2.65^a^	19.2±2.24^c^	10.25±0.42^a^
24 h	1,000	63.82±3.44	54.42±2.53	31.38±4.69^ab^	22.95±2.53^bc^	9.41±1.00^ab^
72 h	0	52.83±0.70^b^	42.84±1.14^b^	20.27±1.06	22.55±1.00	9.99±1.17
72 h	200	61.28±3.89^a^	51.53±4.06^a^	24.77±3.7	26.72±1.05	9.75±0.73
72 h	400	57.01±1.43^ab^	48.89±1.98^ab^	24.01±2.79	24.88±4.00	8.12±0.89
72 h	1,000	55.45±1.37^ab^	47.79±1.31^ab^	20.6±0.80	27.04±1.69	7.66±0.77
120 h	0	44.72±0.50^b^	33.24±1.04^b^	13.21±1.81^b^	20.04±0.88^b^	11.48±1.14
120 h	200	49.38±1.22^a^	37.88±1.39^ab^	12.9±1.45^ab^	24.98±0.27^a^	11.50±0.35
120 h	400	47.31±1.58^ab^	36.42±1.22^ab^	10.62±0.39^ab^	25.8±1.31^a^	10.88±0.64
120 h	1,000	49.61±2.24^a^	38.37±2.11^a^	15.08±0.62^a^	23.29±1.59^ab^	11.24±0.17
168 h	0	41.43±0.37^bc^	31.68±0.89^b^	11.39±1.03^bc^	20.29±1.11^b^	9.75±0.74^ab^
168 h	200	47.21±0.85^a^	38.68±0.87^a^	15.03±1.10^a^	23.63±0.76^a^	8.53±0.86^b^
168 h	400	37.46±2.18^c^	25.94±1.07^c^	8.92±0.40^c^	17.02±0.71^c^	11.52±1.30^a^
168 h	1,000	42.63±1.55^b^	31.44±1.51^b^	12.19±0.44^b^	19.25±1.12^bc^	11.19±0.35^ab^

a–c Different letters indicate significant differences among different concentrations of CPP at the same time (p<0.05).

**Table 2:** Effect of *Codonopsis pilosula* polysaccharide (CPP) on the mitochondrial integrity of sheep spermatozoa (%)

Time (h)	0 mg/L	200 mg/L	400 mg/L	1,000 mg/L
0	94.19±0.93	-	-	-
24	83.84±3.66^b^	91.2±1.39^a^	86.94±1.93^ab^	84.06±0.44^b^
72	83.69±0.88^b^	89.61±1.81^a^	86.16±2.34^ab^	88.84±1.74^ab^
120	65.67±4.51^b^	74.76±2.54^a^	72.87±2.71^ab^	74.74±0.63^a^

a,b Different letters indicate significant differences among different concentrations of CPP at the same time (p<0.05).

**Table 3:** Effect of *Codonopsis pilosula* polysaccharide (CPP) on sperm acrosome integrity in sheep (%)

Time (h)	0 mg/L	200 mg/L	400 mg/L	1 000 mg/L
0	89.29±1.51	-	-	
24	80.65±0.16^b^	87.66±1.26^a^	84.00±2.95^a^	80.52±3.22^ab^
72	71.68±2.54	75.06±2.75	75.75±3.36	75.56±2.44
120	61.61±7.05	71.04±0.50	69.98±0.33	68.71±0.44

a,b Different letters indicate significant differences among different concentrations of CPP at the same time (p<0.05).
